# Acute effects of active breaks during prolonged sitting on subcutaneous adipose tissue gene expression: an ancillary analysis of a randomised controlled trial

**DOI:** 10.1038/s41598-019-40490-0

**Published:** 2019-03-07

**Authors:** Megan S. Grace, Melissa F. Formosa, Kiymet Bozaoglu, Audrey Bergouignan, Marta Brozynska, Andrew L. Carey, Camilla Bertuzzo Veiga, Parneet Sethi, Francis Dillon, David A. Bertovic, Michael Inouye, Neville Owen, David W. Dunstan, Bronwyn A. Kingwell

**Affiliations:** 1 Baker Heart & Diabetes Institute, Melbourne, Australia; 2 Murdoch Children’s Research Institute, and Department of Paediatrics, University of Melbourne, Parkville, VIC Australia; 3 Division of Endocrinology, Metabolism, and Diabetes and Anschutz Health and Wellness Center, University of Colorado, School of Medicine, Aurora, Colorado USA; 4 Institut Pluridisciplinaire Hubert Curien, Université de Strasbourg, CNRS, Strasbourg, France; 5 UMR 7178 Centre National de la Recherche scientifique (CNRS), Strasbourg, France; 6 Department of Public Health and Primary Care, University of Cambridge, Cambridge, CB1 8RN United Kingdom; 7 Swinburne University of Technology, Melbourne, Australia; 8 Mary MacKillop Institute for Health Research, Australian Catholic University, Melbourne, Australia

## Abstract

Active breaks in prolonged sitting has beneficial impacts on cardiometabolic risk biomarkers. The molecular mechanisms include regulation of skeletal muscle gene and protein expression controlling metabolic, inflammatory and cell development pathways. An active communication network exists between adipose and muscle tissue, but the effect of active breaks in prolonged sitting on adipose tissue have not been investigated. This study characterized the acute transcriptional events induced in adipose tissue by regular active breaks during prolonged sitting. We studied 8 overweight/obese adults participating in an acute randomized three-intervention crossover trial. Interventions were performed in the postprandial state and included: (i) prolonged uninterrupted sitting; or prolonged sitting interrupted with 2-minute bouts of (ii) light- or (iii) moderate-intensity treadmill walking every 20 minutes. Subcutaneous adipose tissue biopsies were obtained after each condition. Microarrays identified 36 differentially expressed genes between the three conditions (fold change ≥0.5 in either direction; p < 0.05). Pathway analysis indicated that breaking up of prolonged sitting led to differential regulation of adipose tissue metabolic networks and inflammatory pathways, increased insulin signaling, modulation of adipocyte cell cycle, and facilitated cross-talk between adipose tissue and other organs. This study provides preliminary insight into the adipose tissue regulatory systems that may contribute to the physiological effects of interrupting prolonged sitting.

## Introduction

Prolonged uninterrupted sitting is positively associated with cardiometabolic risk biomarkers and premature mortality, independent of moderate-to-vigorous intensity physical activity^1^. There is emerging interest in the underlying biological responses to prolonged sitting that may drive disease pathophysiology, and how breaking up sitting with short, regular bouts of physical activity can potentially act to mitigate these adverse effects. Studies examining beneficial effects of breaking up sedentary time have implicated regulation of postprandial glucose, insulin and lipid metabolism^2–4^, endothelium-mediated arterial vasodilation^5–7^, and anti-inflammatory mechanisms^8–11^.

The mechanistic underpinnings of the beneficial effects of breaking up prolonged sitting are likely to be multifactorial, and involve peripheral organs that are known to play a key role in metabolism, such as skeletal muscle, liver and adipose tissue. Our group has observed favourable changes in skeletal muscle gene and protein expression that likely contribute to the improved glucose control associated with breaking up of prolonged sitting via light- or moderate-intensity activities. These changes include some which align with, and others which may be distinct from, the effects of continuous acute exercise^12,13^.

Subcutaneous white adipose tissue is another important metabolic regulatory tissue that may be a central mediator of the cardiometabolic effects of breaking up prolonged sitting time. In addition to its key role in lipid storage, factors secreted from white adipose tissue play pivotal roles in appetite regulation, energy homeostasis, insulin sensitivity, inflammation, and immunological responses^14,15^. Excessive accumulation of visceral adipose tissue positively associates with increased cardiometabolic disease risk^16^. While the role of subcutaneous adipose tissue in disease pathophysiology is less clear, it has also been associated with increased disease risk^17–23^.

Storage of excess lipid in subcutaneous adipocytes has been driven by the vital physiological roles of lipids; including as structural elements in plasma membranes, second messengers and energy substrates. However, upon exceeding storage capacity of subcutaneous adipocytes, lipid accumulates in visceral adipose and eventually ectopically in metabolic regulatory organs^17^. Ectopic lipid accumulation has been well established as a contributor to low-grade inflammation, endoplasmic reticulum stress and tissue insulin resistance^17^. In adipose tissue, insulin resistance is characterized by reduced glucose uptake, impairment in both lipogenesis and insulin-stimulated inhibition of lipolysis^14,24^. Thus, insulin resistance increases lipid availability to visceral and ectopic sites, contributing to increased cardiometabolic risk^14^.

Acute exercise protects against ectopic lipid accumulation by oxidising fatty acids for ATP production and enhancing insulin sensitivity in multiple tissues. In adipose tissue, exercise training also reduces inflammation and increases glucose uptake through pathways which have been well characterized^25^. To date, studies have typically focused on understanding the impact of continuous exercise bouts on gene and protein expression. Few studies have investigated whether regular active breaks from prolonged sitting modulate similar pathways^13,26^, and the effects of breaks from prolonged sitting on adipose tissue are unknown. In a previous study we showed that, compared to uninterrupted sitting, frequent brief bouts of either light- or moderate-intensity walking lowered acute postprandial glucose and insulin responses^2^. An ancillary analysis of *vastus lateralis* muscle collected from 8 participants in the main study showed that those brief interruptions to sitting time resulted in upregulation of genes involved in cell development, glucose uptake, and anti-inflammatory pathways; and, downregulation of genes associated with protein degradation and muscle atrophy^13^.

In this investigation, we now aim to define the acute transcriptional events induced in subcutaneous adipose tissue by regular brief active interruptions to prolonged sitting time, using subcutaneous abdominal adipose tissue collected from the same subset of 8 participants in the skeletal muscle-tissue study described above^2,13^. We hypothesized that interrupting sitting time with brief, intermittent activity bouts of either light or moderate intensity would change expression of genes involved in substrate metabolism and inflammation, relative to prolonged sitting.

## Methods

### Study Overview

This randomized, three-intervention crossover trial was conducted in accordance with the Declaration of Helsinki and approved by the Alfred Health Human Research Ethics Committee (Melbourne, Australia). The study is registered with the Australian and New Zealand Clinical Trials Registry (ACTRN12609000656235, 04/08/2009) and participants provided written, informed consent.

**Figure 1:**
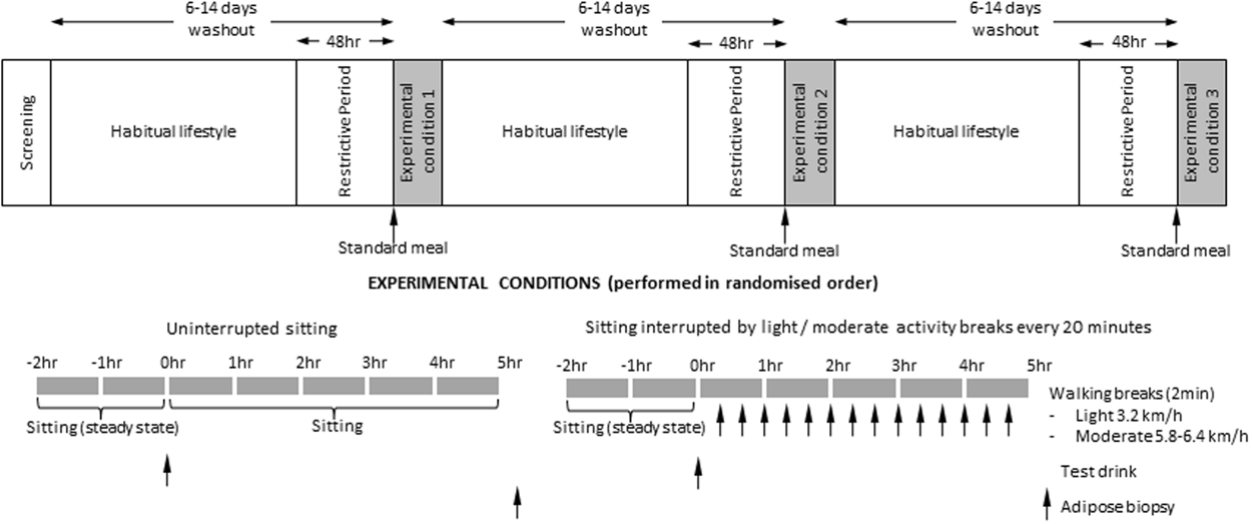
Study design and protocol.

A detailed description of the participant characteristics, screening and testing procedures for the full study have been previously described^2^ and the relevant aspects of the study design and protocol are summarised in Fig. 1. Of the 19 participants in the main study, eight (7 male, 1 female) consented to subcutaneous adipose biopsies, and are included in the current investigation. The inclusion criteria were: age 45–65 years, body mass index (BMI) 25–45 kg/m^2^. Participants were excluded if they had a diagnosis of diabetes, were taking glucose and/or lipid-lowering medication, or were regularly engaged in moderate intensity exercise ≥150 minutes/week for at least 3 months. They attended three study visits at the Baker Heart and Diabetes Institute to complete the study conditions in a randomized order^2^. Study conditions were as follows:

1. *Uninterrupted sitting*. Participants remained seated throughout the experimental period and were instructed to minimize excessive movement, only rising from the chair to void.
2. *Sitting interrupted with light intensity walking breaks*. Participants rose from the seated position every 20 minutes throughout the experimental period (3 breaks per hour) and completed a 2-minute bout of light intensity walking (3.2 km/h) on a motorized treadmill on a level surface. The participants then returned to the seated position. This procedure was undertaken on 14 occasions, providing a total of 28 minutes of light-intensity activity.
3. *Sitting interrupted with moderate intensity walking breaks*. Identical to the light intensity walking breaks condition, but participants completed 2 minute bouts of moderate-intensity walking (5.8–6.4 km/h) on the treadmill, providing a total of 28 minutes of moderate-intensity activity. The speed of walking for this condition was based on individual perception of activity intensity, determined at a familiarisation session, and based on a Borg Rating of Perceived Exertion between 12 and 14^2^.

A minimum washout period of 6 days between each condition was imposed to avoid potential confounding effects since an acute bout of activity may enhance insulin sensitivity for up to 72 hours. Participants were instructed to refrain from any structured moderate-vigorous exercise, alcohol and caffeine in the 48 hours prior to each of the trial conditions. During this time, physical activity was monitored using an Actigraph GT1M accelerometer (Actigraph, Pensacola, FL), which was worn around the hip during waking hours.

Participants reported to the laboratory between 0700 and 0800 hours, having fasted from at least 2200 hours the night before. A cannula was inserted into an antecubital vein for hourly blood sampling. For all of the three experimental conditions, and following the initial blood collection (time point: −2 hours), they remained seated for 2 hours to achieve a steady state before consumption of a standardized test drink (time point: 0 hours). The 200 mL test drink consisted of 75 g carbohydrate (100% corn maltodextrin powder, Natural Health) and 50 g fat (Calogen, Nuticia). The specific nutritional components were as follows: energy: 3,195 kJ; total fat: 50 g; saturated fat: 5 g; monounsaturated fat: 30.4 g; polyunsaturated fat: 14.3 g; total carbohydrate: 75 g; total sugars: 12.8 g; protein nil; fiber <1 g; sodium: 46.9 mg; and water: 90 g. Blood was sampled at baseline before drink consumption and hourly post drink consumption. The incremental area under the glucose-time, insulin-time and insulin/glucose-time curves have been previously presented for the whole cohort^2^, and the 8 participants included in this sub-study^13^.

### Adipose tissue biopsy

Abdominal subcutaneous adipose tissue biopsies were obtained using standard aseptic technique and local anaesthesia (lignocaine) approximately 40–50 minutes after the last activity bout, and 5 hours after the drink ingestion (Fig. 1). During the first intervention visit, a 0.5–1 cm skin incision was made ~5 cm lateral to the navel/umbilicus, and a Bergstrom biopsy needle passed through to obtain approximately 1–2 cm^3^ of subcutaneous adipose tissue under suction. Biopsies taken at the second and third intervention trials were obtained from the side opposite that of the preceding trial and at the third trial >5 cm superior or inferior to the first to avoid the potential for sampling prior injured tissue. All biopsies were rinsed of blood in ice-cold sterile saline, the connective tissue removed and cleaned adipose tissue was snap frozen in liquid nitrogen for subsequent storage at −80 °C until further analysis.

### RNA extraction

RNA isolation from 100 mg of adipose tissue was performed using TRIzol Reagent as per manufacturer’s instructions (ThermoFisher Scientific, Massachusetts, USA). The RNA phase (the clear upper aqueous layer) was transferred to a new tube without disturbing the interphase. Then 1.5 volumes of 100% ethanol was added to the samples and loaded onto the RNeasy mini spin column (Qiagen, Hilden, Germany). RNA was extracted according to the manufacturer’s protocol. The RNA was quantified using a Nanodrop spectrophotometer (ThermoFisher Scientific, Massachusetts, USA) and Qubit fluorometer (ThermoFisher Scientific, Massachusetts, USA). The integrity of the RNA was assessed using a MultiNA Microchip Electrophoresis System (Shimadzu, Kyoto, Japan).

### Gene Expression Profiling/Microarrays

The amplification and labelling of RNA was performed using the TotalPrep amplification kit according to manufacturer’s instructions (Ambion; ThermoFisher Scientific Massachusetts, USA). The labelled samples were hybridized to the Human HT-12 v4.0 Expression BeadChip as per the manufacturer’s protocol (47,231 probes; Illumina, California, USA). The BeadChips were scanned using the iScan microarray BeadStudio platform (Illumina, California, USA). Quality standards for hybridization, labelling, staining, background signal, and basal level of housekeeping gene expression for each chip were verified. After scanning on the Illumina iScan, the resulting images were background subtracted, quantile normalized and processed using the GenomeStudio software (Illumina, California, USA). Data files were deposited into the National Center for Biotechnology Information Gene Expression Omnibus (https://www.ncbi.nlm.nih.gov/geo/query/acc.cgi?acc = GSE115645).

An Illumina detection P-value of <0.05 was used to determine presence or absence of a probe in each tissue sample. Call rates for each of the three study conditions were then calculated for each gene, per condition using equation 1:

1 $$Call\,rate=\frac{number\,of\,adipose\,samples\,with\,a\,detection\,p\,value < 0.05}{total\,number\,of\,adipose\,samples}$$

A gene was included in the subsequent analysis if a call rate of ≥0.5 was calculated for at least one condition. Analysis of the effects of activity breaks versus prolonged sitting was performed in Stata (StataCorp, College Station, TX). To identify genes with the most marked changes in expression level, we applied an absolute fold change threshold of ≥0.5 (in either direction from 1) for any of the three experimental condition pairs (light intensity breaks *versus* uninterrupted sitting; moderate intensity breaks *versus* uninterrupted sitting; moderate *versus* light intensity breaks), where a fold change <1 refers to downregulation and >1 refers to upregulation of the gene relative to the comparator condition. This approach has been previously validated^27^, showing close qualitative and quantitative relationships to qPCR, including in skeletal muscle from this same study^13^.

### Statistical Analysis

Linear mixed models accounting for dependency in the data (repeated measures) were used to evaluate the differential effects of the trial conditions on expression of genes. All models were adjusted for age and BMI. P-values for the overall condition variable were obtained from post-hoc Wald tests and corrected for multiple comparisons using the False Discovery Rate (FDR) method of Benjamini-Hochberg^28^. *Post hoc*, pairwise analyses were performed using post-estimation commands of the linear mixed model, and the resultant pairwise P-values were corrected by the Dunn-Sidak approach. A corrected P-value of <0.05 was considered to be statistically significant. All statistical analyses were performed using Stata 14.1 for Windows (StataCorp, College Station, TX).

### Pathway analysis

Genes were ranked using a signed log_10_-transformed P-value (from the linear mixed model analysis) with the sign denoting the direction of change, positive for increasing and negative for decreasing^29^. The rank score for genes that were represented by more than one transcript, was calculated for the transcript with the lowest P-value, and the others were disregarded. The ranked gene list was inputted into GSEA 3.0 software (Broad Institute, Cambridge, MA). A normalized enrichment score (NES), the degree to which a gene set is overrepresented at the top or bottom of a ranked list of genes, was calculated^30^. NES scores greater than zero indicate upregulation of a pathway, whereas NES values of less than zero represent down regulation.

Gene sets with a P-value ≤ 0.1, following false discovery rate (FDR) correction, were considered likely to generate valid hypotheses and drive further research. Leading edge analysis was used to examine the genes that were in the leading-edge subsets of the enriched gene sets. The Reactome pathway database (www.reactome.org) was used to categorize genes by pathways to facilitate biological interpretation.

All analyses were performed in a blinded manner.

## Results

Participant demographics and biochemical analyses for the eight participants recruited for this sub-study have been reported previously^13^. Those recruited for the present sub-study had a mean age of 55 ± 6 years and BMI 30.9 ± 2.9 kg/m^2^ (mean ± SD). In the main study (*n* = 19), interrupting prolonged sitting with both light- and moderate-intensity walking significantly reduced postprandial glucose (by 24% and 30%, respectively) and insulin (by 23% for both conditions) incremental area under the curve (iAUC), relative to uninterrupted sitting^2^. In the current subset of eight participants, there was a trend toward a decrease in glucose iAUC (−22% and −20%; *P* = 0.1) and a significant decrease in insulin iAUC (−25% and −23%; *P* < 0.05) in the light-intensity and moderate-intensity breaks conditions, respectively, relative to uninterrupted sitting^13^.

**Figure 2:**
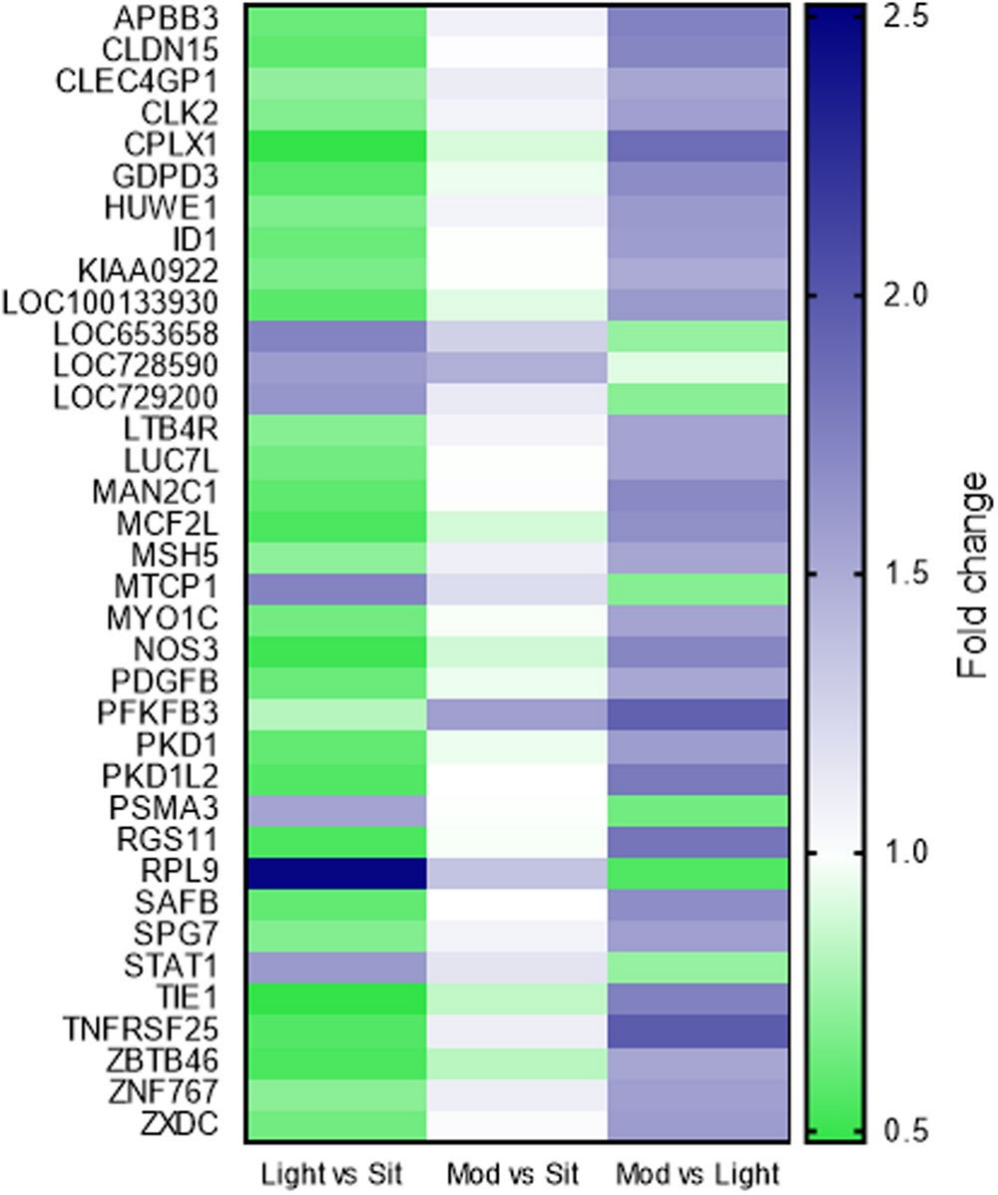
Genes differentially expressed between the three experimental conditions. Heat map showing those genes up (blue) and down (green) regulated for comparisons of Light vs Sit (light intensity breaks versus uninterrupted sitting, column 1); Mod vs Sit (moderate intensity breaks versus uninterrupted sitting, column 2) and Mod vs Light (moderate intensity breaks versus light intensity breaks, column 3). Comparisons meeting both the 0.5 fold change criteria (in either direction from 1) and the significance threshold are highlighted in Table 1. Refer to Table 1 for gene definitions.

The microarray analysis identified 18,844 transcripts expressed in the adipose tissue. Of these, 469 satisfied the 0.5-fold change criteria (described in the Methods) in at least one out of the three comparisons between the experimental condition pairs. Thirty-six genes were significantly differentially expressed (FDR-adjusted P < 0.05; Table 1, Fig. 2). Of these, 7/36 genes were upregulated and 2/36 genes were downregulated in the light-intensity breaks condition, compared to uninterrupted sitting; 1/36 genes was upregulated and none were downregulated in the moderate-intensity breaks condition compared to uninterrupted sitting; and, 29/36 genes were upregulated and none were downregulated in the moderate-intensity breaks condition compared to the light-intensity breaks (Table 1).

**Table 1:** Genes differentially expressed between the three experimental conditions.

Gene Symbol	Overall condition	Light vs Sit		Mod vs Sit		Mod vs Light		Definition
	P Value	Fold Change	P Value	Fold Change	P Value	Fold Change	P Value	
APBB3	0.037	0.62	0.001	1.08	0.92	**1**.**74**	**0**.**0001**	Amyloid beta precursor protein binding family member 3
CLDN15	0.044	0.59	0.0006	1.01	1.00	**1**.**72**	**0**.**0004**	Claudin 15
CLEC4GP1	0.033	0.72	0.004	1.11	0.67	**1**.**53**	<**0**.**0001**	C-type lectin domain family 4 member G pseudogene 1
CLK2	0.0008	0.68	<0.0001	1.07	0.84	**1**.**57**	<**0**.**0001**	CDC like kinase 2
CPLX1	0.030	**0**.**48**	**0**.**0001**	0.90	0.91	**1**.**87**	**0**.**001**	Complexin 1
GDPD3	0.006	0.57	<0.0001	0.95	0.97	**1**.**68**	**0**.**0001**	Glycerophosphodiester phosphodiesterase domain containing 3
HUWE1	0.001	0.67	<0.0001	1.07	0.84	**1**.**60**	<**0**.**0001**	HECT, UBA and WWE domain containing 1, E3 ubiquitin protein ligase
ID1	0.004	0.62	<0.0001	0.99	1.00	**1**.**58**	<**0**.**0001**	Inhibitor of DNA binding 1, dominant negative helix-loop-helix protein
KIAA0922	0.011	0.66	<0.0001	0.99	1.00	**1**.**50**	**0**.**0001**	Transmembrane 131 like
LOC100133930	0.0005	0.58	<0.0001	0.92	0.84	**1**.**60**	<**0**.**0001**	Similar to D-2-hydroxyglutarate dehydrogenase
LOC653658	0.006	**1**.**73**	<**0**.**0001**	1.27	0.092	0.73	0.016	Ribosomal protein S23 pseudogene 8
LOC728590	0.030	**1**.**59**	<**0**.**0001**	1.47	0.002	0.92	0.86	Ribosomal protein S27a pseudogene 11
LOC729200	0.006	**1**.**62**	<**0**.**0001**	1.13	0.56	0.70	0.001	Small nuclear ribonucleoprotein polypeptide B2 pseudogene
LTB4R	0.030	0.69	0.001	1.06	0.92	**1**.**54**	**0**.**0001**	Leukotriene B4 receptor
LUC7L	0.032	0.64	0.0003	0.99	1.00	**1**.**54**	**0**.**0004**	Luciferase 7 like
MAN2C1	0.008	0.59	<0.0001	1.01	1.00	**1**.**70**	<**0**.**0001**	Mannosidase alpha class 2C member 1
MCF2L	0.008	0.54	<0.0001	0.89	0.78	**1**.**66**	**0**.**0006**	MCF2 transforming sequence-like protein
MSH5	0.001	0.71	0.0002	1.09	0.68	**1**.**53**	<**0**.**0001**	MutS homolog 5
MTCP1	0.027	**1**.**73**	<**0**.**0001**	1.20	0.38	0.69	0.008	Mature T-cell proliferation 1
MYO1C	0.0003	0.64	<0.0001	0.98	1.00	**1**.**54**	<**0**.**0001**	Myosin 1c
NOS3	0.013	0.51	<0.0001	0.88	0.80	**1**.**72**	**0**.**0009**	Nitric oxide synthase 3
PDGFB	0.015	0.62	<0.0001	0.95	0.94	**1**.**52**	**0**.**0004**	Platelet derived growth factor subunit b
PFKFB3	0.029	0.81	0.42	**1**.**57**	**0**.**008**	**1**.**94**	<**0**.**0001**	6-phosphofructo-2-kinase/fructose-2,6-biphosphatase 3
PKD1	0.0003	0.60	<0.0001	0.95	0.94	**1**.**58**	<**0**.**0001**	Polycystin 1, transient receptor potential channel interacting
PKD1L2	0.0005	0.56	<0.0001	1.00	1.00	**1**.**79**	<**0**.**0001**	Polycystin 1 like 2
PSMA3	0.0002	**1**.**54**	<**0**.**0001**	0.99	1.00	0.64	<0.0001	Proteasome subunit alpha 3
RGS11	0.045	0.54	0.0004	0.98	1.00	**1**.**82**	**0**.**0006**	Regulator of g protein signalling 11
RPL9	0.008	**2**.**48**	<**0**.**0001**	1.35	0.30	0.55	0.004	Ribosomal protein L9
SAFB	0.023	0.60	0.0002	1.00	1.00	**1**.**67**	**0**.**0002**	Scaffold attachment factor b
SPG7	0.040	0.68	0.002	1.07	0.92	**1**.**57**	**0**.**0002**	Spastic paraplegia 7, paralegin matrix AAA peptidase subunit
STAT1	0.010	**1**.**60**	<**0**.**0001**	1.16	0.36	0.73	0.004	Signal transducer and activator of transcription 1
TIE1	0.001	**0**.**48**	<**0**.**0001**	0.84	0.53	**1**.**75**	**0**.**0003**	Tyrosine kinase with immunoglobulin like and EGF like domains 1
TNFRSF25	0.0005	0.56	<0.0001	1.10	0.83	**1**.**97**	<**0**.**0001**	Tumour necrosis factor receptor superfamily member 25
ZBTB46	0.047	0.54	<0.0001	0.82	0.46	**1**.**53**	**0**.**011**	Zinc finger and BTB domain containing 46
ZNF767	0.030	0.70	0.002	1.10	0.75	1.57	<0.0001	Zinc finger family member 767, pseudogene
ZXDC	0.0008	0.64	<0.0001	1.02	1.00	**1**.**59**	<**0**.**0001**	ZXD family zinc finger C

Overall condition P value indicates the corrected false discovery rate P value for the post-hoc Wald test. Pairwise condition comparison P values are corrected by the Dunn-Sidak approach. Comparisons meeting the 0.5 fold change criteria (in either direction) and meeting the significance threshold are highlighted in bold: fold change comparisons < 1 indicate downregulation, and >1 upregulation of the gene relative to the comparison condition. Light vs Sit = light intensity breaks versus uninterrupted sitting; Mod vs Sit = moderate intensity breaks versus uninterrupted sitting; Mod vs Light = moderate intensity breaks versus light intensity breaks.

**Figure 3:**
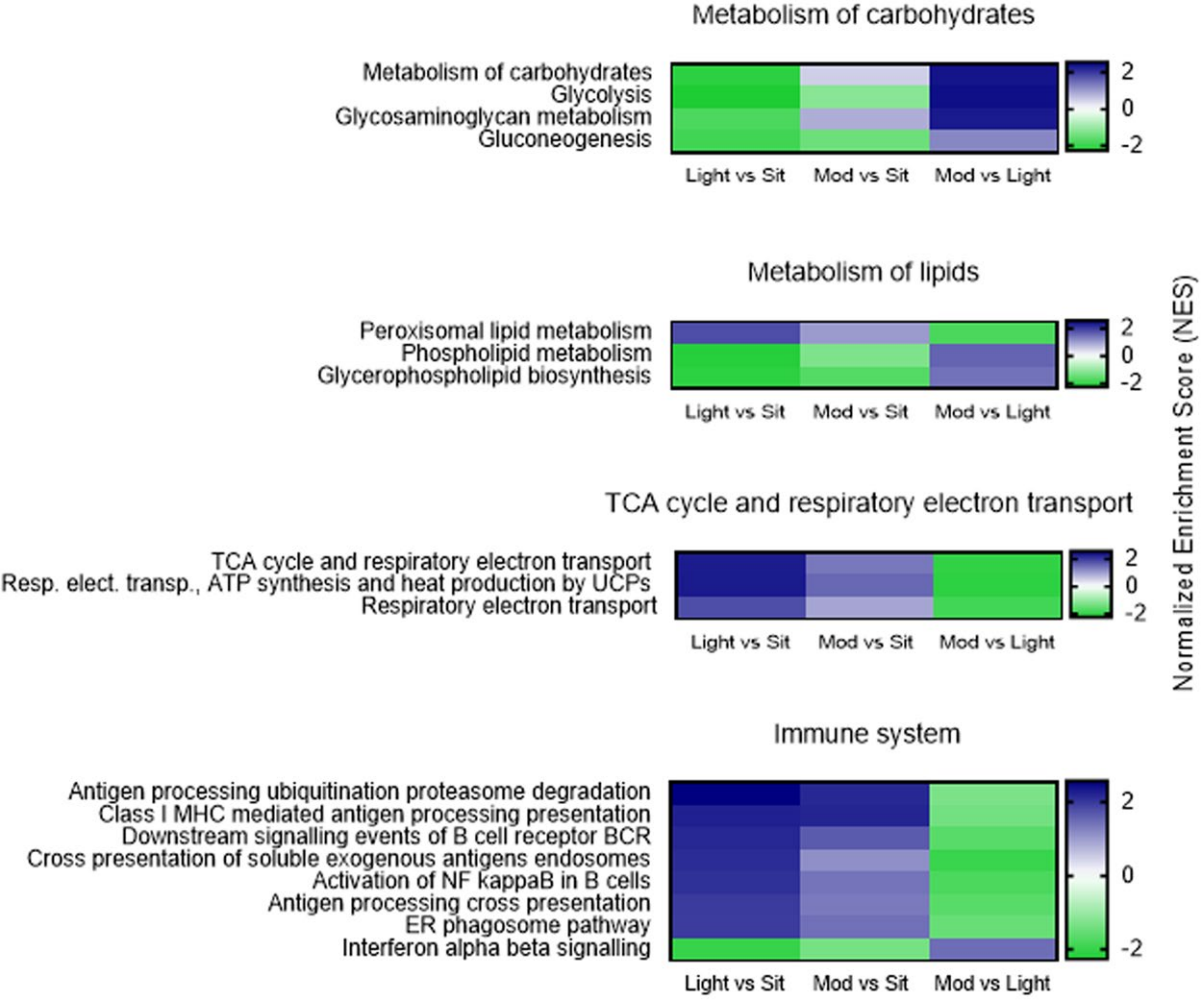
Pathways differentially regulated between the three experimental conditions. Heat map showing pathways up (blue, Normalized Enrichment Score (NES) >0) and down (green, NES <0) regulated for comparisons of Light vs Sit (light intensity breaks versus uninterrupted sitting, column 1); Mod vs Sit (moderate intensity breaks versus uninterrupted sitting, column 2) and Mod vs Light (moderate intensity breaks versus light intensity breaks, column 3). The significance of these comparisons are indicated in Supplementary Table 1. ATP = adenosine triphosphate; BCR = B cell receptor; ER = endoplasmic reticulum; MHC = major histocompatibility; Resp. electr. transp = respiratory electron transport; TCA = tricarboxcylic acid.

Analysis of the ranked gene list identified 102 differentially (up- or down-) regulated pathways, with an FDR-adjusted P ≤ 0.10 (Fig. 3, Supplementary Table 1). In the light-intensity breaks condition, 64/102 pathways were upregulated and 19/102 were downregulated, compared to uninterrupted sitting. In the moderate-intensity breaks condition, 11/102 pathways were upregulated and 0/102 were downregulated, compared to uninterrupted sitting; and, 23/102 pathways were upregulated and 42/102 were downregulated, compared to light-intensity breaks. Among the regulated pathways were those involved with metabolism of macronutrients, adenosine triphosphate (ATP) synthesis, immune function, signal transduction, extracellular matrix organisation and cell cycle.

## Discussion

The major novel finding of this study is that actively breaking up prolonged sitting time in the postprandial state was associated with changes in the expression of subcutaneous abdominal adipose tissue genes and pathways involved in macronutrient metabolism and ATP synthesis, immune function, signal transduction, and cell cycle regulation. These changes are likely induced by systemic physiological drivers associated with physical activity and known to modulate adipose tissue function through multiple mechanisms including via the sympathetic nervous system, increased adipose tissue blood flow and changes in circulating factors including adrenaline, insulin and glucagon^31^. Interestingly, the majority of the differences were between the light-intensity activity, relative to both the uninterrupted sitting and the moderate-intensity breaks conditions. This may relate to the fact that of all the interventions studied, light intensity breaks would be most dependent on fat metabolism (adipose tissue)^32,33^. By contrast, moderate intensity breaks which would have been more reliant on glucose metabolism, had relatively few differentially regulated genes and pathways in comparison to prolonged uninterrupted sitting.

### Active breaks during prolonged sitting alter adipose expression of metabolic regulatory networks controlling ATP synthesis

Consistent with fat oxidation being the major pathway for ATP production induced by low-intensity activity, upregulation of genes and pathways associated with enhanced capacity for lipid oxidation were observed for the light-intensity breaks condition in comparison to both the uninterrupted sitting and moderate-intensity breaks conditions (Figs 2 and 3). Mitochondrial (tricarboxylic acid cycle and respiratory electron transport) and peroxisomal β-oxidation pathways were upregulated in the light-intensity breaks condition. The key functional difference between these compartmental processes is that mitochondrial β-oxidation generates acetyl-CoA and reduced cofactors via degradation of energy substrates, primarily long-chain fatty acids, which is coupled to oxidative phosphorylation; whereas, peroxisomes degrade a wide variety of lipophilic compounds that have important functions not involved in ATP resynthesis^34^. Although it did not reach the fold-change cut-off for the main analysis (1.45 fold change compared to uninterrupted sitting), upregulation of the NDUFAB1 (NADH:Ubiquinone Oxidoreductase Subunit AB1) gene was identified in the pathway analysis for the light-intensity breaks condition. This non-catalytic accessory subunit of the mitochondrial membrane Complex I functions in the transfer of electrons from NADH to the respiratory chain, indicating an increased capacity for substrate oxidation. Significantly lower expression of ID1 and NOS3 (endothelial nitric oxide synthase, eNOS) in the light-intensity breaks condition, compared to the uninterrupted sitting and moderate-intensity breaks conditions, is also consistent with higher lipid oxidation. Deletion of the ID1 gene (a transcriptional regulator of helix-loop-helix transcription factors that control cell type-specific gene expression) in mice has been implicated in numerous physiological effects with potential metabolic benefit. It was found that loss of the ID1 gene leads to increases in expression of genes which promote fatty acid oxidation, with *id1*^*−/−*^ mice also exhibiting reduced insulin resistance and fat mass while on a high fat diet, and increased oxygen consumption compared to wild type mice^35,36^. Adipose eNOS expression is increased in obese adults, and has been implicated in the inhibition of lipolysis via downregulation of lipolytic pathways^37,38^. Downregulation of eNOS (NOS3) expression in the light-intensity breaks condition may therefore also indicate an increase in lipolysis. The differing expression patterns between the two break conditions for genes regulating lipolysis occurred despite similarity in blood glucose and insulin levels, suggesting that mechanisms associated with activity break intensity such as adrenergic pathways may have been the predominant influence.

Reflecting the preponderance of lipid over carbohydrate metabolism for provision of ATP during light-intensity activity physical activity^32,33^, pathways linked to carbohydrate metabolism were downregulated in comparison to both uninterrupted sitting and moderate-intensity breaks. Here, significantly lower adipose tissue NOS3 and MYO1C gene expression in the light-intensity breaks condition may indicate reduced insulin-dependent and -independent GLUT-4 translocation and subsequent glucose uptake^39,40^. In contrast, significantly higher expression of PFKFB3 was observed in the moderate-intensity breaks condition compared to both the uninterrupted sitting and light-intensity breaks conditions. PFKFB3 is an enzyme whose product (fructose 2,6-biphosphate) is a powerful activator of the glycolytic enzyme 6-phosphofructo-1-kinase, the rate-limiting step in glycolysis^41,42^, indicating a greater capacity for glucose oxidation in the moderate-intensity breaks condition (Fig. 3).

### Active breaks in prolonged sitting regulate anti-inflammatory and anti-oxidative stress pathways, and improve insulin sensitivity

Inflammatory and metabolic signaling are closely interlinked, such that chronic disturbance of metabolic homeostasis can lead to aberrant immune responses and inflammation; and, vice versa, where chronic inflammation can induce insulin resistance and metabolic dysfunction^43^. While a continuous moderate-to-high intensity exercise bout has been shown to activate inflammatory and oxidative pathways^44,45^, we found little evidence for modulation of immune function or inflammatory status with the brief moderate-intensity breaks from sitting in the current study, compared to uninterrupted sitting (Fig. 3). In contrast, light intensity breaks from sitting appeared to upregulate immune function and downregulate inflammatory signals compared to the uninterrupted sitting and moderate-intensity breaks conditions.

Tumour necrosis factor alpha (TNFα), derived primarily from resident macrophages, is known to be overexpressed in adipose tissue of obese humans and mice^46–48^. TNFα is a pro-inflammatory cytokine that activates various signal transduction cascades, including pathways involved in inhibiting insulin action^43,49,50^. TNFα and other inflammatory cytokines impair insulin action by post-translational modification of insulin receptor substrate 1 through serine phosphorylation, which interferes with the ability of this protein to engage in insulin-receptor signaling^43^. The ID1 gene has been suggested to be important in mediating the increased expression of TNFα in adipose tissue following a high-fat diet in mice^36^; the ID1 gene was downregulated in the light-intensity breaks compared to the moderate-intensity breaks condition. Receptors for TNFα play an important role in its downstream effects. Here, we also observed lower expression of the receptor TNFRSF25 in the light-intensity breaks condition; TNFRSF25 is known to stimulate NF-κB activity^51^. NF-κB inflammatory pathways contribute to the pathology of metabolic disorders^52^. Indeed, clinical trials have shown amelioration of insulin resistance and improved glucose homeostasis in type 2 diabetes patients treated with salicylates, which inhibit NF-κB activation^53^. Significantly lower expression of ID1 and TNFRSF25 in the light-intensity breaks condition compared to moderate-intensity breaks is suggestive of reduced production of TNFα and its downstream signaling. Despite not reaching the 1.5 fold change threshold, both the ID1 and TNFRSF25 genes were also significantly downregulated in the light-intensity breaks condition compared to uninterrupted sitting.

The potent pro-inflammatory molecule LTB4 is highly expressed in obesity, and has recently been shown to directly induce insulin resistance at least partially through its receptor, LTB4R^54–56^. Here, we observed significantly lower expression of the LTB4R gene in the light-intensity breaks, compared to the moderate-intensity breaks condition. LTB4R was also statistically significantly downregulated in the light-intensity breaks condition compared to uninterrupted sitting though the 0.5 fold change threshold was not reached (0.69 fold change). Furthermore, the Notch signaling pathway was significantly downregulated in the light-intensity breaks condition, compared to uninterrupted sitting and moderate-intensity breaks conditions. Notch signaling is highly conserved in mammals, and rodent studies indicate that inhibition of this pathway improves adipose tissue insulin sensitivity^57^. Together, these results suggest that downregulation of inflammatory pathways involving TNFα, NF-κB, lipoxygenase and Notch signaling could contribute to the beneficial effects of breaking up sedentary time with light-intensity activities on metabolism by positively influencing adipose tissue insulin sensitivity.

Reactive oxygen species (ROS) are continuously produced as by-products of aerobic metabolism. ROS can act as important signaling molecules, but can also cause oxidative damage to cells and tissues, and their accumulation is regulated by a complex system of anti-oxidative defences. In addition to β-oxidation, the peroxisomal lipid metabolism pathway is involved in the dynamic response to cellular stress. Peroxisomes contain a number of antioxidant defences involved in the regulation of ROS, including the synthesis of the ether phospholipid species plasmalogens^58,59^. We have previously shown that light-intensity breaks during prolonged sitting in adults with type 2 diabetes ameliorates the postprandial reduction in plasma plasmalogen species, compared to prolonged uninterrupted sitting^4^. The upregulation of peroxisomal lipid metabolism in the light-intensity breaks condition in the current study suggests that this may be partially due to increased biosynthesis of plasmalogen lipid species in the adipose tissue. This finding may provide an important mechanistic link between the protective effects of light-intensity breaks during prolonged sitting and risk of cardiometabolic diseases, as plasmalogens have been suggested to be protective against atherosclerosis^60,61^.

### Active breaks in prolonged sitting regulate adipose tissue cell cycle pathways

Coupling of cell cycle progression and programmed cell death pathways are regulated to maintain tissue homeostasis. The cell cycle involves a cascade of events that leads to cell division and DNA replication. Progression of the cell cycle is orchestrated by regulatory (cyclins) and catalytic subunits (cyclin-dependent kinases, CDKs), as well as ubiquitin ligases (such as anaphase promoting complex, APC) which mark cell cycle proteins for degradation^62,63^. Here, we observed upregulation of several pathways related to cell cycle and DNA replication, concurrently with upregulation of apoptosis pathways, in response to light-intensity breaks from sitting versus uninterrupted sitting. These data suggest adaptation of adipocyte cell cycle in the light-intensity breaks condition. Cell cycle and DNA replication pathways also tended to be upregulated in the moderate-intensity breaks condition, in comparison to uninterrupted sitting, but in most cases did not reach statistical significance (P > 0.1, Table 2). Upregulation of the M, G1, and S phases of cell cycle involved in cell growth and proliferation, as well as pathways involved in the transition between these phases, indicates acute stimulation of adipogenesis pathways. Upregulation of checkpoints also suggests greater quality control to prevent unhealthy cells from proliferating^62^.

Adipose cells dynamically adapt their growth and metabolism to current requirements, and crosstalk between these two biological responses has recently been identified^64^. Although relatively few studies have been conducted in humans, animal models provide strong evidence that, in addition to the control of cell proliferation and death, cell cycle mediators also play key roles in the biological function of adipocytes such as insulin sensitivity, lipolysis and glucose transport^62,63^. Therefore, acute upregulation of cell cycle pathways with active breaks in prolonged sitting (independently of break intensity) may indicate a role for these effectors in metabolic adaptation of adipose tissue to physical activity. This is also characteristic of other general cellular function pathways (e.g. metabolism of RNA and proteins, DNA replication) which were also upregulated with breaks at both intensities.

### Light intensity breaks in prolonged sitting may facilitate cross-talk between adipose and other organs

The configuration of adipose tissue is structured so that adipocytes are in close proximity to immune cells, with immediate access to a vast network of blood vessels, which allows continuous and dynamic interaction between immune and metabolic processes and cross-talk with other organs^43^. This structure and inter-connectivity likely contributes to the key role of adipose tissue in the development of metabolic disease^43^. Indeed, an active communication network between adipose tissue and muscle regulating glucose homeostasis is now well-established, whereby adipokines can increase muscle insulin sensitivity and glucose uptake via direct and indirect means^65,66^. Wnt glycoproteins are produced and released from several tissues, including white adipose tissue. Wnt signaling inhibits adipogenesis and regulates whole-body metabolism by altering the behaviour of multiple cell types and tissues, including a role in promoting insulin sensitivity^66,67^. Moreover, Wnt signaling activation in adipose progenitors has been shown to promote insulin-independent muscle glucose uptake^65^. Therefore, in the current study upregulation of the Wnt signaling pathway in the light-intensity breaks condition, compared to uninterrupted sitting, could indicate facilitation of cross-talk between the adipose tissue and other body organs.

### Strengths and limitations

Our highly controlled study design, including within-participant comparison across three interventions completed in a randomized order, is a strength. The number of participants, albeit relatively small, was sufficient to detect significant changes in gene expression with appropriate FDR correction. A larger cohort may reveal a greater number of regulated genes. Similarly, we investigated only the acute effects of 5 hours of light- and moderate-intensity breaks from sitting and chronic interventions (multiple days/weeks) may show evolution in gene expression patterns. Differential effects are likely to be observed in adipose tissue collected from other subcutaneous (eg, gluteal) or visceral depots, although there is no evidence for site specific relationships in terms of substrate provision to local active muscle (spot reduction)^68^. It is also relevant to consider the potential contribution of RNA derived from immune and endothelial cells within our adipose tissue samples. Generally, such components are unlikely to have contributed significantly to our observations. Adipose samples used in the present analysis were dissected free of connective and vascular tissue, and washed thoroughly minimizing any vascular component. Immune cells would however remain^69^, and future studies dissecting their potential contribution to metabolism during breaks from prolonged sitting will be important. The absolute fold-change criteria of 0.5 (in either direction) for selection of significant effects may result in failure to detect important regulated genes; however, all genes meeting the call rate criteria were included in the pathway analysis and therefore contributed to the overall biological interpretation of the results. False positives were minimized by use of repeated measures analysis to allow comparison of all three interventions, and correction for multiple testing with the FDR method by Benjamini-Hochberg^28^. Our findings provide a basis for future studies to examine the adipose tissue transcriptional networks, in various adipose depots, regulated by breaking up sedentary time over longer periods and their relationship to bioclinical phenotypes. In particular, studies with a larger sample size and detailed measurements of glucose and fat metabolism beyond the simple plasma measurements employed in the current study will be necessary to substantiate the findings of the present analysis.

## Conclusions

This is the first study to characterize the acute transcriptional events induced in subcutaneous abdominal adipose tissue by breaking up prolonged sitting time with frequent brief activity bouts. We observed distinct and biologically-interpretable effects of what conventionally would be understood as an extremely modest physical activity stimulus. Interestingly, upregulation of pathways involved in oxidative metabolism and immunity were greater for light-intensity breaks compared to sitting, although moderate-intensity breaks showed similar directional trends. Our findings highlight some of the regulated mechanisms that potentially underlie the physiological benefits of breaking up prolonged periods of sedentary time. We have identified candidate genes that may contribute to the modulationof postprandial glucose, insulin, lipid and inflammatory responses shown to occur with breaking up of prolonged sitting. These findings further elucidate the potential mechanistic underpinnings of the adverse health consequences of prolonged sitting. They add further weight to the scientific literature describing the potential health effects of regular, brief activity breaks in sitting (even of a light intensity), which could contribute to the development and extension of future public health guidelines. However larger studies with more detailed metabolic measures are required to corroborate the findings of the current investigation.

## Supplementary information

Supplementary Dataset 1
