# Supplementary material for: Acute effects of active breaks during prolonged sitting on subcutaneous adipose tissue gene expression: an ancillary analysis of a randomised controlled trial
Source: Sci Rep. 2019 Mar 7;9:3847. doi: 10.1038/s41598-019-40490-0 (PMC6405989; doi:10.1038/s41598-019-40490-0)
Supplement: Supplementary file 1 — Supplementary Dataset 1 [file 41598_2019_40490_MOESM1_ESM.docx]

**Acute effects of active breaks during prolonged sitting on subcutaneous adipose tissue gene expression:**

an ancillary analysis of a randomised controlled trial

Megan S. Grace^1^, Melissa F. Formosa^1^, Kiymet Bozaoglu^1,2^, Audrey Bergouignan^3,4,,5^, Marta Brozynska^1,6^, Andrew L. Carey^1^, Camilla Bertuzzo Veiga^1^, Parneet Sethi^1^, Francis Dillon^1^, David A. Bertovic^1^, Michael Inouye^1,6^, Neville Owen^1 ,7^, David W. Dunstan^1^, Bronwyn A. Kingwell^1^**^*^**

^1^Baker Heart & Diabetes Institute, Melbourne, Australia

^2^Murdoch Children’s Research Institute, and Department of Paediatrics, University of Melbourne, Parkville, VIC, Australia

^3^Division of Endocrinology, Metabolism, and Diabetes and Anschutz Health and Wellness Center, University of Colorado, School of Medicine, Aurora, Colorado, USA

^4^Institut Pluridisciplinaire Hubert Curien, Université de Strasbourg, CNRS, Strasbourg, France.

^5^UMR 7178 Centre National de la Recherche scientifique (CNRS), Strasbourg, France.

^6^ Department of Public Health and Primary Care, University of Cambridge, Cambridge CB1 8RN, United Kingdom

^7^ Swinburne University of Technology, Melbourne, Australia

*Supplementary Table 1. Differentially regulated biological pathways between the three experimental conditions*

| **Pathway** | **Comparison** | **NES** | **P-value** | **No. of Genes** |
| --- | --- | --- | --- | --- |
| *Metabolism of carbohydrates* |  |  |  |  |
| Metabolism of carbohydrates | Light vs Sit | -2.12 | 0.060 | 78 |
|  | Mod vs Sit | 0.48 | 1.00 |  |
|  | Mod vs Light | 2.35 | 0.013 | 47 |
| Glycolysis | Light vs Sit | -2.27 | 0.102 |  |
|  | Mod vs Sit | -1.19 | 0.673 |  |
|  | Mod vs Light | 2.40 | 0.010 | 62 |
| Glycosaminoglycan metabolism | Light vs Sit | -1.80 | 0.132 |  |
|  | Mod vs Sit | 0.81 | 0.901 |  |
|  | Mod vs Light | 2.29 | 0.015 | 44 |
| Gluconeogenesis | Light vs Sit | -1.91 | 0.091 | 17 |
|  | Mod vs Sit | -1.47 | 1.00 |  |
|  | Mod vs Light | 1.20 | 0.451 |  |
| *Metabolism of lipids* |  |  |  |  |
| Peroxisomal lipid metabolism | Light vs Sit | 1.78 | 0.064 | 15 |
|  | Mod vs Sit | 1.00 | 0.715 |  |
|  | Mod vs Light | -1.82 | 0.084 | 40 |
| Phospholipid metabolism | Light vs Sit | -2.18 | 0.074 | 74 |
|  | Mod vs Sit | -1.29 | 0.679 |  |
|  | Mod vs Light | 1.55 | 0.278 |  |
| Glycerophospholipid biosynthesis | Light vs Sit | -2.11 | 0.058 | 36 |
|  | Mod vs Sit | -1.72 | 0.925 |  |
|  | Mod vs Light | 1.41 | 0.346 |  |
| *TCA cycle and respiratory electron transport* |  |  |  |  |
| TCA cycle and respiratory electron transport | Light vs Sit | 2.27 | 0.006 | 67 |
|  | Mod vs Sit | 1.35 | 0.446 |  |
|  | Mod vs Light | -2.06 | 0.035 | 26 |
| Respiratory electron transport, ATP synthesis by chemiosmotic coupling, and heat production by uncoupling proteins | Light vs Sit | 2.29 | 0.005 | 38 |
|  | Mod vs Sit | 1.51 | 0.383 |  |
|  | Mod vs Light | -2.11 | 0.027 | 24 |
| Respiratory electron transport | Light vs Sit | 1.77 | 0.065 | 38 |
|  | Mod vs Sit | 0.90 | 0.800 |  |
|  | Mod vs Light | -1.90 | 0.067 | 31 |
| *Metabolism of amino acids and derivatives* |  |  |  |  |
| Metabolism of amino acids and derivatives | Light vs Sit | 2.24 | 0.007 | 96 |
|  | Mod vs Sit | 1.91 | 0.162 |  |
|  | Mod vs Light | -0.79 | 0.918 |  |
| Regulation of ornithine decarboxylase ODC | Light vs Sit | 2.32 | 0.004 | 39 |
|  | Mod vs Sit | 1.63 | 0.327 |  |
|  | Mod vs Light | -1.86 | 0.076 | 36 |
| *Metabolism of proteins* |  |  |  |  |
| Metabolism of proteins | Light vs Sit | 3.60 | <0.0001 | 177 |
|  | Mod vs Sit | 1.63 | 0.332 |  |
|  | Mod vs Light | -2.61 | 0.001 | 21 |
| SRP-dependent co-translational protein targeting to membrane | Light vs Sit | 5.01 | <0.0001 | 79 |
|  | Mod vs Sit | 2.32 | 0.043 | 84 |
|  | Mod vs Light | -4.35 | <0.0001 | 9 |
| Translation | Light vs Sit | 4.96 | <0.0001 | 97 |
|  | Mod vs Sit | 2.71 | 0.001 | 74 |
|  | Mod vs Light | -4.03 | <0.0001 | 14 |
| Peptide chain elongation | Light vs Sit | 4.35 | <0.0001 | 67 |
|  | Mod vs Sit | 1.86 | 0.182 |  |
|  | Mod vs Light | -4.08 | <0.0001 | 11 |
| Formation of the ternary complex and subsequently the 43S complex | Light vs Sit | 3.67 | <0.0001 | 35 |
|  | Mod vs Sit | 1.63 | 0.343 |  |
|  | Mod vs Light | -2.67 | 0.001 | 21 |
| Activation of the mRNA upon binding of the CAP binding complex and EIFS and subsequent binding to 43S | Light vs Sit | 3.53 | <0.0001 | 40 |
|  | Mod vs Sit | 1.72 | 0.296 |  |
|  | Mod vs Light | -2.30 | 0.010 | 23 |
| Mitochondrial protein import | Light vs Sit | 1.75 | 0.070 | 24 |
|  | Mod vs Sit | 0.97 | 0.746 |  |
|  | Mod vs Light | -1.41 | 0.314 |  |
| Unfolded protein response | Light vs Sit | -1.92 | 0.090 | 36 |
|  | Mod vs Sit | 0.98 | 0.734 |  |
|  | Mod vs Light | 1.99 | 0.060 | 25 |
| Activation of chaperone genes by XBP1S | Light vs Sit | -2.64 | 0.018 | 28 |
|  | Mod vs Sit | -0.96 | 0.873 |  |
|  | Mod vs Light | 2.70 | 0.002 | 136 |
| Activation of genes by ATF4 | Light vs Sit | 0.70 | 0.910 |  |
|  | Mod vs Sit | -0.77 | 0.942 |  |
|  | Mod vs Light | -2.16 | 0.022 | 24 |
| *Metabolism of RNA* |  |  |  |  |
| Metabolism of RNA | Light vs Sit | 4.46 | <0.0001 | 124 |
|  | Mod vs Sit | 2.17 | 0.061 | 72 |
|  | Mod vs Light | -3.38 | <0.0001 | 16 |
| Nonsense mediated decay enhanced by the exon junction complex | Light vs Sit | 4.26 | <0.0001 | 66 |
|  | Mod vs Sit | 1.87 | 0.179 |  |
|  | Mod vs Light | -3.24 | <0.0001 | 17 |
| Regulation of mRNA stability by proteins that bind AU rich elements | Light vs Sit | 2.51 | 0.001 | 30 |
|  | Mod vs Sit | 1.36 | 0.445 |  |
|  | Mod vs Light | -1.90 | 0.066 | 31 |
| Destabilization of mRNA by AUF1 HNRNP D0 | Light vs Sit | 2.23 | 0.007 | 20 |
|  | Mod vs Sit | 1.43 | 0.407 |  |
|  | Mod vs Light | -1.69 | 0.125 |  |
| Deadenylation dependent mRNA decay | Light vs Sit | 1.86 | 0.043 | 18 |
|  | Mod vs Sit | 1.38 | 0.429 |  |
|  | Mod vs Light | -1.62 | 0.150 |  |
| *Metabolism of nucleotides* |  |  |  |  |
| Metabolism of nucleotides | Light vs Sit | 1.65 | 0.104 |  |
|  | Mod vs Sit | -0.80 | 0.930 |  |
|  | Mod vs Light | -1.78 | 0.087 | 42 |
| Synthesis and interconversion of nucleotide di and triphosphates | Light vs Sit | 0.88 | 0.743 |  |
|  | Mod vs Sit | -1.25 | 0.681 |  |
|  | Mod vs Light | -2.17 | 0.022 | 24 |
| *Signal transduction* |  |  |  |  |
| Signalling by WNT | Light vs Sit | 2.32 | 0.004 | 33 |
|  | Mod vs Sit | 1.24 | 0.492 |  |
|  | Mod vs Light | -1.62 | 0.152 |  |
| Insulin receptor recycling | Light vs Sit | 1.93 | 0.030 | 11 |
|  | Mod vs Sit | 1.52 | 0.394 |  |
|  | Mod vs Light | -1.22 | 0.488 |  |
| Signalling by NOTCH1 | Light vs Sit | -1.94 | 0.090 | 27 |
|  | Mod vs Sit | 1.27 | 0.486 |  |
|  | Mod vs Light | 1.82 | 0.126 |  |
| Signalling by NGF | Light vs Sit | -2.07 | 0.066 | 79 |
|  | Mod vs Sit | 1.34 | 0.453 |  |
|  | Mod vs Light | 2.84 | 0.001 | 150 |
| GPCR downstream signalling | Light vs Sit | -2.03 | 0.063 | 101 |
|  | Mod vs Sit | -1.29 | 0.665 |  |
|  | Mod vs Light | 2.21 | 0.023 | 33 |
| Signalling by GPCR | Light vs Sit | -2.04 | 0.063 | 123 |
|  | Mod vs Sit | -1.35 | 0.742 |  |
|  | Mod vs Light | 2.45 | 0.011 | 91 |
| Signalling by NOTCH | Light vs Sit | -2.06 | 0.062 | 51 |
|  | Mod vs Sit | 1.11 | 0.614 |  |
|  | Mod vs Light | 2.22 | 0.024 | 38 |
| Cell death signalling via NRAGE NRIF and NADE | Light vs Sit | -1.09 | 0.619 |  |
|  | Mod vs Sit | 1.26 | 0.487 |  |
|  | Mod vs Light | 2.41 | 0.011 | 90 |
| p75 NTR receptor mediated signalling | Light vs Sit | -1.14 | 0.587 |  |
|  | Mod vs Sit | 1.07 | 0.646 |  |
|  | Mod vs Light | 2.07 | 0.048 | 28 |
| NRAGE signals death through JNK | Light vs Sit | -1.35 | 0.395 |  |
|  | Mod vs Sit | 0.65 | 0.952 |  |
|  | Mod vs Light | 2.06 | 0.049 | 27 |
| NGF signalling via TRKA from the plasma membrane | Light vs Sit | -1.72 | 0.152 |  |
|  | Mod vs Sit | 1.24 | 0.491 |  |
|  | Mod vs Light | 2.03 | 0.056 | 27 |
| Pre NOTCH expression and processing | Light vs Sit | -1.78 | 0.133 |  |
|  | Mod vs Sit | 1.09 | 0.623 |  |
|  | Mod vs Light | 2.01 | 0.058 | 26 |
| Signalling by RHO GTPases | Light vs Sit | -1.50 | 0.314 |  |
|  | Mod vs Sit | -1.27 | 0.665 |  |
|  | Mod vs Light | 1.92 | 0.086 | 24 |
| *Extracellular matrix organisation* |  |  |  |  |
| Extracellular Matrix Organisation | Light vs Sit | -2.16 | 0.069 | 29 |
|  | Mod vs Sit | 1.22 | 0.489 |  |
|  | Mod vs Light | 2.64 | 0.003 | 120 |
| Collagen formation | Light vs Sit | -1.94 | 0.087 | 21 |
|  | Mod vs Sit | 0.90 | 0.794 |  |
|  | Mod vs Light | 2.32 | 0.014 | 45 |
| *Cell cycle* |  |  |  |  |
| Cell cycle | Light vs Sit | 2.38 | 0.003 | 89 |
|  | Mod vs Sit | 2.11 | 0.070 | 65 |
|  | Mod vs Light | -1.34 | 0.367 |  |
| Mitotic M M G1 Phases | Light vs Sit | 2.86 | <0.0001 | 54 |
|  | Mod vs Sit | 2.40 | 0.074 | 116 |
|  | Mod vs Light | -2.03 | 0.038 | 27 |
| Regulation of mitotic cell cycle | Light vs Sit | 2.61 | 0.001 | 38 |
|  | Mod vs Sit | 1.13 | 0.598 |  |
|  | Mod vs Light | -2.09 | 0.030 | 25 |
| Cell cycle mitotic | Light vs Sit | 2.60 | 0.001 | 83 |
|  | Mod vs Sit | 2.23 | 0.051 | 79 |
|  | Mod vs Light | -1.31 | 0.394 |  |
| Cell cycle checkpoints | Light vs Sit | 2.54 | 0.001 | 36 |
|  | Mod vs Sit | 1.50 | 0.396 |  |
|  | Mod vs Light | -1.31 | 0.397 |  |
| APC C CDC20 mediated degradation of mitotic proteins | Light vs Sit | 2.54 | 0.001 | 34 |
|  | Mod vs Sit | 1.25 | 0.492 |  |
|  | Mod vs Light | -2.02 | 0.040 | 28 |
| APC C CDH1 mediated degradation of CDC20 and other APC C CDH1 targeted proteins in late mitosis early G1 | Light vs Sit | 2.51 | 0.001 | 33 |
|  | Mod vs Sit | 1.18 | 0.532 |  |
|  | Mod vs Light | -2.01 | 0.040 | 28 |
| Assembly of the pre-replicative complex | Light vs Sit | 2.45 | 0.002 | 43 |
|  | Mod vs Sit | 1.56 | 0.386 |  |
|  | Mod vs Light | -1.85 | 0.078 | 37 |
| Autodegradation of CDH1 by CDH1 APC C | Light vs Sit | 2.44 | 0.002 | 31 |
|  | Mod vs Sit | 1.35 | 0.444 |  |
|  | Mod vs Light | -1.71 | 0.116 |  |
| M G1 transition | Light vs Sit | 2.43 | 0.002 | 27 |
|  | Mod vs Sit | 1.74 | 0.299 |  |
|  | Mod vs Light | -1.75 | 0.099 | 43 |
| P53 dependent G1 DNA damage response | Light vs Sit | 2.41 | 0.002 | 43 |
|  | Mod vs Sit | 1.82 | 0.220 |  |
|  | Mod vs Light | -1.53 | 0.212 |  |
| Autodegradation of the E3 ubiquitin ligase COP1 | Light vs Sit | 2.32 | 0.004 | 39 |
|  | Mod vs Sit | 1.65 | 0.347 |  |
|  | Mod vs Light | -1.84 | 0.080 | 38 |
| ORC1 removal from chromatin | Light vs Sit | 2.29 | 0.005 | 45 |
|  | Mod vs Sit | 1.56 | 0.373 |  |
|  | Mod vs Light | -1.82 | 0.082 | 40 |
| Cyclin E associated events during G1 S transition | Light vs Sit | 2.25 | 0.006 | 32 |
|  | Mod vs Sit | 1.40 | 0.425 |  |
|  | Mod vs Light | -1.89 | 0.067 | 34 |
| G1 S transition | Light vs Sit | 2.24 | 0.007 | 35 |
|  | Mod vs Sit | 1.52 | 0.381 |  |
|  | Mod vs Light | -1.65 | 0.138 |  |
| CDK mediated phosphorylation and removal of CDC6 | Light vs Sit | 2.22 | 0.007 | 37 |
|  | Mod vs Sit | 1.46 | 0.412 |  |
|  | Mod vs Light | -1.83 | 0.079 | 39 |
| S Phase | Light vs Sit | 2.21 | 0.007 | 35 |
|  | Mod vs Sit | 1.55 | 0.361 |  |
|  | Mod vs Light | -1.37 | 0.343 |  |
| CDT1 association with the CDC6 ORC origin complex | Light vs Sit | 2.19 | 0.008 | 37 |
|  | Mod vs Sit | 1.27 | 0.483 |  |
|  | Mod vs Light | -1.99 | 0.042 | 29 |
| Mitotic G1 G1 S Phases | Light vs Sit | 2.19 | 0.008 | 51 |
|  | Mod vs Sit | 1.34 | 0.447 |  |
|  | Mod vs Light | -1.27 | 0.428 |  |
| SCFSKP2 mediated degradation of P27 P21 | Light vs Sit | 2.17 | 0.008 | 29 |
|  | Mod vs Sit | 1.39 | 0.423 |  |
|  | Mod vs Light | -2.04 | 0.039 | 27 |
| P53 independent G1 S DNA damage checkpoint | Light vs Sit | 2.17 | 0.009 | 26 |
|  | Mod vs Sit | 1.36 | 0.445 |  |
|  | Mod vs Light | -1.80 | 0.085 | 41 |
| SCF beta TRCP mediated degradation of EMI1 | Light vs Sit | 2.16 | 0.009 | 27 |
|  | Mod vs Sit | 1.14 | 0.593 |  |
|  | Mod vs Light | -2.12 | 0.027 | 27 |
| Synthesis of DNA | Light vs Sit | 2.16 | 0.009 | 29 |
|  | Mod vs Sit | 1.63 | 0.324 |  |
|  | Mod vs Light | -1.33 | 0.380 |  |
| Mitotic prometaphase | Light vs Sit | 1.9 | 0.034 | 26 |
|  | Mod vs Sit | 2.01 | 0.106 |  |
|  | Mod vs Light | -1.25 | 0.449 |  |
| APC CDC20 mediated degradation of NEK2A | Light vs Sit | 1.74 | 0.074 | 34 |
|  | Mod vs Sit | 1.28 | 0.489 |  |
|  | Mod vs Light | -0.62 | 0.979 |  |
| Recruitment of mitotic centrosome proteins and complexes | Light vs Sit | -1.77 | 0.127 |  |
|  | Mod vs Sit | 1.23 | 0.494 |  |
|  | Mod vs Light | 1.90 | 0.094 | 24 |
| *Programmed cell death* |  |  |  |  |
| Regulation of apoptosis | Light vs Sit | 1.99 | 0.022 | 20 |
|  | Mod vs Sit | 1.58 | 0.373 |  |
|  | Mod vs Light | -1.66 | 0.138 |  |
| *Immune system* |  |  |  |  |
| Antigen processing ubiquitination proteasome degradation | Light vs Sit | 2.56 | 0.001 | 92 |
|  | Mod vs Sit | 2.15 | 0.062 | 69 |
|  | Mod vs Light | -1.32 | 0.389 |  |
| Class I MHC mediated antigen processing presentation | Light vs Sit | 2.23 | 0.007 | 95 |
|  | Mod vs Sit | 2.18 | 0.064 | 76 |
|  | Mod vs Light | -1.41 | 0.310 |  |
| Downstream signalling events of B cell receptor BCR | Light vs Sit | 2.16 | 0.009 | 51 |
|  | Mod vs Sit | 1.64 | 0.352 |  |
|  | Mod vs Light | -1.67 | 0.133 |  |
| Cross presentation of soluble exogenous antigens endosomes | Light vs Sit | 2.11 | 0.012 | 35 |
|  | Mod vs Sit | 1.12 | 0.605 |  |
|  | Mod vs Light | -1.99 | 0.043 | 30 |
| Activation of NF kappaB in B cells | Light vs Sit | 2.07 | 0.014 | 33 |
|  | Mod vs Sit | 1.39 | 0.424 |  |
|  | Mod vs Light | -1.80 | 0.085 | 41 |
| Antigen processing cross presentation | Light vs Sit | 1.97 | 0.024 | 33 |
|  | Mod vs Sit | 1.33 | 0.459 |  |
|  | Mod vs Light | -1.67 | 0.131 |  |
| ER phagosome pathway | Light vs Sit | 1.95 | 0.027 | 29 |
|  | Mod vs Sit | 1.43 | 0.408 |  |
|  | Mod vs Light | -1.52 | 0.216 |  |
| Interferon alpha beta signalling | Light vs Sit | -2.01 | 0.067 | 35 |
|  | Mod vs Sit | -1.37 | 0.758 |  |
|  | Mod vs Light | 1.45 | 0.320 |  |
| *DNA replication* |  |  |  |  |
| DNA Replication | Light vs Sit | 2.63 | 0.001 | 57 |
|  | Mod vs Sit | 2.27 | 0.048 | 83 |
|  | Mod vs Light | -1.79 | 0.086 | 42 |
| Assembly of the pre-replicative complex | Light vs Sit | 2.45 | 0.002 | 43 |
|  | Mod vs Sit | 1.56 | 0.386 |  |
|  | Mod vs Light | -1.85 | 0.078 | 37 |
| M G1 transition | Light vs Sit | 2.43 | 0.002 | 27 |
|  | Mod vs Sit | 1.74 | 0.299 |  |
|  | Mod vs Light | -1.75 | 0.099 | 43 |
| ORC1 removal from chromatin | Light vs Sit | 2.29 | 0.005 | 45 |
|  | Mod vs Sit | 1.56 | 0.373 |  |
|  | Mod vs Light | -1.82 | 0.082 | 40 |
| CDK mediated phosphorylation and removal of CDC6 | Light vs Sit | 2.22 | 0.007 | 37 |
|  | Mod vs Sit | 1.46 | 0.412 |  |
|  | Mod vs Light | -1.83 | 0.079 | 39 |
| CDT1 association with the CDC6 ORC origin complex | Light vs Sit | 2.19 | 0.008 | 37 |
|  | Mod vs Sit | 1.27 | 0.483 |  |
|  | Mod vs Light | -1.99 | 0.042 | 29 |
| Synthesis of DNA | Light vs Sit | 2.16 | 0.009 | 29 |
|  | Mod vs Sit | 1.63 | 0.324 |  |
|  | Mod vs Light | -1.33 | 0.380 |  |
| *Developmental biology* |  |  |  |  |
| Developmental Biology | Light vs Sit | -2.19 | 0.084 | 91 |
|  | Mod vs Sit | 0.93 | 0.799 |  |
|  | Mod vs Light | 2.93 | 0.001 | 139 |
| Semaphorin interactions | Light vs Sit | -1.94 | 0.094 | 24 |
|  | Mod vs Sit | -1.05 | 0.816 |  |
|  | Mod vs Light | 1.67 | 0.214 |  |
| Axon guidance | Light vs Sit | -2.16 | 0.061 | 64 |
|  | Mod vs Sit | -0.81 | 0.931 |  |
|  | Mod vs Light | 2.35 | 0.014 | 49 |
| *Transport of small molecules* |  |  |  |  |
| Iron uptake and transport | Light vs Sit | 1.93 | 0.030 | 13 |
|  | Mod vs Sit | 1.18 | 0.527 |  |
|  | Mod vs Light | -1.57 | 0.183 |  |
| Transferrin endocytosis and recycling | Light vs Sit | 1.84 | 0.046 | 10 |
|  | Mod vs Sit | 1.25 | 0.501 |  |
|  | Mod vs Light | -1.10 | 0.638 |  |
| Metal ion SLC transporters | Light vs Sit | 1.77 | 0.064 | 12 |
|  | Mod vs Sit | -0.88 | 0.962 |  |
|  | Mod vs Light | -1.56 | 0.186 |  |
| ABC family proteins mediated transport | Light vs Sit | -1.74 | 0.142 |  |
|  | Mod vs Sit | -0.85 | 0.942 |  |
|  | Mod vs Light | 2.16 | 0.031 | 30 |

*Italicized indicates higher-level pathway, non-italicized indicates subcategory pathway. P value corrected for false discovery rate by Benjamini Hochberg method. Significant (p<0.1) comparisons are highlighted: positive NES (normalized enrichment score) value indicates upregulated (blue) and negative NES value indicates downregulated (green) pathway compared to the uninterrupted sitting condition. No. of genes indicates the number of associated genes contributing to the enrichment score. Light vs Sit = light intensity breaks versus uninterrupted sitting; Mod vs Sit = moderate intensity breaks versus uninterrupted sitting; Mod vs Light = moderate intensity breaks versus light intensity breaks.*
